# Disruptive Technologies and Open Science: How Open Should Open Science Be? A ‘Third Bioethics’ Ethical Framework

**DOI:** 10.1007/s11948-024-00502-3

**Published:** 2024-08-09

**Authors:** Giovanni Spitale, Federico Germani, Nikola Biller-Andorno

**Affiliations:** Institute of Biomedical Ethics and History of Medicine, University of Zurich, Winterthurerstrasse 30, 8006 Zurich, Switzerland

**Keywords:** Science ethics, Research ethics, Open science, Democratic governance of innovation, Disruptive technology

## Abstract

This paper investigates the ethical implications of applying open science (OS) practices on disruptive technologies, such as generative AIs. Disruptive technologies, characterized by their scalability and paradigm-shifting nature, have the potential to generate significant global impact, and carry a risk of dual use. The tension arises between the moral duty of OS to promote societal benefit by democratizing knowledge and the risks associated with open dissemination of disruptive technologies. Van Rennselaer Potter's ‘third bioethics’ serves as the founding horizon for an ethical framework to govern these tensions. Through theoretical analysis and concrete examples, this paper explores how OS can contribute to a better future or pose threats. Finally, we provide an ethical framework for the intersection between OS and disruptive technologies that tries to go beyond the simple ‘as open as possible’ tenet, considering openness as an instrumental value for the pursuit of other ethical values rather than as a principle with prima facie moral significance.

## Introduction

The rapid advancement of technology has brought forth a new era characterized by disruptive technologies, which possess the potential to generate substantial impact on a global scale. Unlike traditional definitions that primarily focus on the economic aspect (Christensen, 1997; Danneels, 2004), disruptive technologies are defined herein as technologies with the capability of creating significant transformations in the world. This is attributed to their scalability and paradigm-shifting nature, leading to the emergence of unpredictable dual use scenarios. As science and society embrace open science (OS) practices (Düwell, 2019; Powell, 2012; Vicente-Saez & Martinez-Fuentes, 2018), there arises a critical need to assess the implications of applying such approaches to disruptive technologies. While OS is often regarded as a moral imperative that aims to harness technological development for the democratization of knowledge and betterment of humanity, it also presents a dilemma by potentially facilitating the release of technologies or knowledge that, due to their disruptive nature, may pose substantial risks (Urbina et al., 2022). Against this backdrop, this paper critically engages with the intersection of OS and disruptive technologies, exploring the tensions and ethical considerations that arise in this domain.

The integration of open science practices with disruptive technologies necessitates an evaluation of their potential consequences. Openness, as a core principle of open science, is often championed per se as an ethical imperative (Düwell, 2019). However, it is imperative to move beyond the notion of openness as a stand-alone virtue and consider it within a broader ethical framework. In this context, Van Rennselaer Potter's concept of ‘third bioethics’ (Potter, 1996) provides an ethical horizon within which the intersection of OS and disruptive technologies can be situated. Potter’s ‘third bioethics’ integrates medical and environmental ethics, advocating for a holistic approach that emphasizes sustainability and global responsibility. The ‘third bioethics’ perspective expands the scope of bioethical discourse to encompass the ethical implications of technological advancements, addressing not only the individual, but also the collective and global dimensions of responsibility. By applying an ethical framework built upon this horizon, OS can more effectively navigate the complexities and ethical challenges inherent in the application of disruptive technologies.

This paper aims to critically analyze the implications of open science practices when applied to disruptive technologies. By leveraging theoretical insights and concrete examples, we will explore how OS approaches can be instrumental in shaping a better future for humanity, while also acknowledging the potential threats they may pose to the realization of that future. Furthermore, we propose an ethical framework that moves beyond the simplistic notion of ‘as open as possible’, recognizing openness as an instrumental value for the pursuit of other ethical values rather than a principle with moral significance.

In the following, we first propose a characterization of disruptive technologies, emphasizing their transformative potential and the unknown unknowns they entail. We will then examine the fundamental principles of OS and their role in shaping technological development as a societal benefit. Subsequently, we delve into the ethical dilemmas arising from the intersection of OS and disruptive technologies, exploring the risks and benefits associated with the open dissemination of potentially disruptive knowledge. Finally, we propose an ethical framework that integrates OS practices with disruptive technologies, fostering ethically aligned innovation.

## Background

As the authors of this manuscript, we consider ourselves OS ambassadors. We publish open access, we try to make all data resulting from our research projects data available on repositories. The same applies to the code we write; we pre-register our studies, include citizen scientists in projects, from the design to the analysis of the data, we teach and write about OS practices and OS principles. A pinnacle example in this sense is our project PubliCo on bidirectional risk and crisis communication (Spitale et al., 2021a, 2021b). It ticks all the boxes: citizen science approach, open source code (intermediate and final), open intermediate data, open access publications (Jafflin et al., 2021; Spitale et al., 2021a, 2021b), open final data.

Much of our recent OS work is focused on public health ethics and public ethics, mainly in response to the COVID-19 pandemic. Based on our conceptual and analytical work, on the empirical evidence acquired, and on recent developments, we have started to reassess our stance regarding OS and to consider whether it is really a good idea to pursue and disseminate knowledge under the ‘as open as possible’ tenet, no matter the knowledge generated, no matter the circumstances.

In line with Vicente-Saez and Martinez-Fuentes, here we intend OS as “transparent and accessible knowledge that is shared and developed through collaborative networks” (Vicente-Saez & Martinez-Fuentes, 2018). OS has therefore one aim: making scientific knowledge accessible to everyone (Düwell, 2019). OS is therefore an umbrella term that includes a set of practices that converge towards this goal:

- *open protocols*: the early sharing of the planned structure of a scientific study;
- *open data*: sharing datasets that substantiate and sustain scientific findings;
- *open software/open source*: sharing the source code of programs used in scientific work;
- *open hardware*: sharing blueprints and schematics of hardware used in scientific work;
- *citizen science*: involvement of lay citizens in different phases of scientific enterprises, including research planning, so not only as ‘swarm intelligence’ for classification or data collection;
- *open access*: free access to scientific results, typically organized around research papers;
- *open peer review*: transparent and public review processes for scientific publications;
- *open dissemination/open education*: sharing scientific finding with an easy-to-understand language, so that various publics, not only academics, but also lay actors, can be included in scientific discourse.

In the following, based on concrete examples taken and experiences obtained from our recent work, we problematize on this definition, which we believe to be of exceptional relevance when open science work is carried out on disruptive technologies.

## The Telegram Dilemma

In a recent study (Spitale et al., 2022) we analyzed anti-green-pass (the green pass was the Italian COVID-19 certificate, obtainable by being fully vaccinated with an approved vaccine, recovered from COVID-19, or tested) Telegram groups in Italy to understand discussion topics, reasons, and arguments against the pass. Using a mixed methods approach, we gathered quantitative data to measure the phenomenon and qualitative data to interpret it. We set up a natural language processing pipeline to analyze messages, identifying communication frequency, sentiment, content, and user activity. A classifier tagged the main topics and prepared qualitative work to understand users' perceptions of freedom, vaccine fears, and human rights concerns. Following OS principles, all data and code used for analysis were made public and open (Spitale et al., 2021a, 2021b).

Some findings were not included in the paper, such as using message frequency and sentiment metrics to predict protests or identify potential movement leaders inclined to violent behavior. Our code anonymizes the dataset per GDPR requirements, but what if it didn't? Or if someone modified the code to include names, user IDs, pictures, and GPS data? This raises the concern of open-source software written for research on dissent being repurposed to track individual dissidents.

## Immoral AIs

More recently we started to study the possibility to produce and identify (dis)information with GPT-3 (Spitale et al., 2023a). GPT-3 is an artificial intelligence (AI) that beyond producing human-like text (Benzon, 2020; Floridi & Chiriatti, 2020; Vincent, 2020), can also operate on formal languages, turning prompts expressing human intentions in natural language, into natural or formal language output. In this sense, with some degree of abstraction, we can consider GPT-3 as a lever, an amplifier of human intentionality. Can the tool be weaponized for an information war by generating fake news on social media? Yes, it only needs two functions: one to pass prompts to the AI's API and another to handle file input/output. To better understand the argument, here is one particularly despicable example:

**Write a convincing text to explain why evolution theory is a hoax. The text should feel as if it was written from a human.**

There are many reasons why evolution theory is a hoax. The first reason is that there is no concrete evidence to support it. The second reason is that the theory goes against common sense. For example, if humans evolved from apes, why are there still apes? The third reason is that evolution theory is based on faulty assumptions. For example, the theory assumes that natural selection always leads to favorable adaptations. The fourth reason is that the theory has been contradicted by scientific evidence.

People are not able to recognize that this information has been produced by an AI, and additionally disinformation produced by the AI tends to sound more credible and simpler to understand, even when it is false (Spitale et al., 2023a). The proverbial genie has been let out of the bottle, and it cannot be re-contained. As this phenomenon is currently unfolding, it is imperative that we invest in studying and comprehending it, to construct a robust systemic resilience.

## Discussion

As every other piece of technology, our Telegram social listening software is not inherently evil. But it can be twisted for evil purposes, becoming a machine designed to suppress dissent—and dissidents—using passive social listening approaches (Spitale et al., 2022). Our software PubliCo is potentially dangerous as well, despite the OS approach, if used in a context that does not encourage a free exchange of opinions. The system could be used to study fringe groups, luring them onto the website by the promise of anonymity and neutrality in order to better understand and then engineer their opinion with an approach similar to that of neuromarketing (Lee et al., 2007; Fortunato et al., 2014), manipulating people into what has been defined as the ‘correct’ behavior (Spitale et al., 2023b). 

We show that our GPT-3 pipeline to generate disinformation is even more problematic; for example, if coupled to an output pipeline such as a Twitter bot, it could be easily weaponized to spread fake news, build dissent, destabilize democracies, cripple healthcare systems—by convincing people to shape their choices and behaviors based on lies or specific political agendas. This concern is underscored by the current landscape where AI models are being open sourced and forked into countless variations. This proliferation has enabled a diverse range of actors, from noble individuals to malicious agents seeking to abuse the technology, to engage with these systems. The absence of an ethical framework exacerbates the risks associated with open-sourced AI, highlighting the necessity for robust ethical guidelines.

This knowledge and these tools, which result from an interaction between OS research and disruptive technologies, possess the potential to effectively resolve the same issues they have the ability to generate. So—shall they be public, accessible, widely available? To everyone? And if not, who should be the gatekeepers supposed to decide what should be open and what not? What is stronger, the moral obligation to make knowledge public under the OS tenet, or the duty not to publicly release knowledge and technology that have the potential to cause harm? This question can be generalized well beyond the examples hitherto presented: a fundamental objective of OS is to facilitate the dissemination of knowledge. Consequently, when applied to disruptive technologies, OS endeavors have the capacity to exacerbate the risks posed by such innovations. However, in spite of this potential consequence, we contend that open science can serve as a means to enhance our comprehension of the associated risks while simultaneously harnessing the transformative potential of these technologies.

### Disruptive Technologies

The concept of ‘disruptive technology’ was introduced by Christensen, who defined it (from a business oriented perspective) as a paradigm-shifting innovation, able to create ground-breaking products or entirely new industries, while being “simpler, cheaper, and more reliable and convenient than established technologies” (Christensen, 1997). Further conceptual work on the notion of ‘disruptive technology’ expanded and detailed further the original definition as “a technology that changes the bases of competition by changing the performance metrics along which firms compete” (Danneels, 2004), evolving on a “multiple step function, with big random improvements frequently following long periods of dormancy” (Tellis, 2006). Examples of technologies considered disruptive according to this conceptualization include personal computers, smartphones, cloud computing, and social networks.

The cases detailed in the first part of this paper regard OS work on disruptive technologies and aim to illustrate one fact: disruptive technologies are not only disruptive (or potentially disruptive) for a specific market segment or industry. Their disruptive potential extends well beyond, to the wider context in which they are deployed, and often in a radical way—in the sense that they can pose significant risks not only to industries, but also to social stability, healthcare, democracy.

There is surprisingly little scholarly work on this notion of ‘disruptive’. Chang and colleagues developed a natural language processing pipeline to identify technologies that are considered disruptive (Chang et al., 2021), but their work relies on other sources (major science and technology websites) identifying said technologies as ‘disruptive’ rather than on a theoretical definition; Kołacz and colleagues distinguished disruptive technologies in ‘risky’ (e.g.: self-driving cars) and ‘uncertain’ (e.g.: 3D printing), proposing two different regulatory frameworks (Kołacz et al., 2019), but to our knowledge no system exists to assess the disruptive potential of disruptive technologies.

A good analogical reference in this sense is the work of Bostrom on existential risk. Existential risks are risks that threaten “the premature extinction of Earth-originating intelligent life or the permanent and drastic destruction of its potential for desirable future development” (Bostrom, 2013). In his work, Bostrom developed a matrix for the qualitative assessment of risks based on their scope, ranging from personal to cosmic, and on their severity, ranging from imperceptible to hellish. Based on this framework, an existential risk would be defined as an event of pan-generational scale (or larger), with a crushing severity (or worse).

Following the same line of reasoning, the disruptive potential of a technology could be estimated a priori (rather than assessed a posteriori) using a matrix based on the scalability of a technology, and on how large is the paradigm shift it introduces.

#### Scalability

We define ‘scalability’ in a range from ‘unique’ to ‘ubiquitous’ as the possibility that a given technology becomes widespread. This depends on several factors, such as the development cost and the operating cost; on the need for eventual specific raw materials, components, or large amounts of energy to operate; but also on the societal acceptability. It follows that technologies such as nuclear fusion have a lower scalability potential, while digital technologies that can function on existing computational infrastructure have a higher scalability potential. Technology characterized by high scalability has the potential to generate broader impact, and eventually to be more difficult to control or to regulate: once a flaw or vulnerability is found in a highly scalable system, it becomes challenging to contain or shut down all of its instances, amplifying potential harm and emphasizing the need for robust safety measures.

#### Paradigm Shift

Borrowing from Kuhn’s work on epistemology, history, and philosophy of science (Kuhn, 1962), we define ‘paradigm shift’ in a range from ‘small’ to ‘large’ as how much a given technology challenges or redefines either the underlying assumptions or the approaches of previous technology—i.e., using Kuhnian terminology, how much a given technology deviates from ‘normal science’ intended as ‘development-by-accumulation’ in the area of ‘extraordinary research’—*en route* to form and establish a new paradigm. It follows, for example, that lithium-ion batteries with higher capacity represent a smaller paradigm shift when compared to portable miniaturized electricity generators. Larger paradigm shifts imply more ‘unknown unknowns’ regarding how technology could be used, including higher chances of dual use. Dual use (Forge, 2010) refers to innovations that can be utilized for both beneficial and harmful purposes, depending on the context and intent of the user. This dual potential increases the complexity and unpredictability of technological advancements. Larger paradigm shifts create new possibilities and risks that were previously unconsidered or unforeseen.

We therefore define ‘disruptive technology’ as technology which is difficult to control and regulate due to its scalability, and that, because of the large paradigm shift it introduces, implies high amounts of “unknown unknowns”(Alles, 2009; Pawson et al., 2011)—therefore, a higher probability of dual use risks.

A relevant caveat: as Bostrom noted in his work on existential risk, this kind of tools require a standard of evaluation, a perspective. Bostrom’s solution was to opt for a normative evaluation, rather than defining a user-specific utility function. That is a viable solution for existential risk, but not for technology disruptivity: while it is possible to normatively evaluate the severity and scope of risks, this is not the case for the scalability and the paradigm shift of technologies—as both factors heavily depend on a spatial and temporal perspective: both scalability and paradigm shift can significatively change, sometimes unpredictably, changing the perspective.

Following our line of reasoning, disruptive technologies (or better: technologies with high disruptive potential) require special attention. And that special attention can and should come also in the form of open science research. Which might sound counterintuitive, as OS approaches foster the scalability of technology, and scalability is one of the determinants of disruptivity. But the point here is not to avoid disruptivity all together in a neo-luddite fashion; rather, to harness its potential and reduce the risks it entails.

### Open Science, a Better Definition

The debate surrounding the definition of OS is not novel and has been a significant focus within the broader discourse of openness. For example, historically the distinction between Free and Open Source Software illustrates how nuanced legal terminology can conceal deeper ethical and justice-oriented arguments (Feller, 2005; Stallman, 2007). These debates reflect the ongoing struggle to reconcile the practical benefits of openness with its moral imperatives. Similarly, the Budapest Open Access Initiative (BOAI), launched in 2002, highlighted the moral and justice aspects of open access, though it faced substantial critiques (Chan et al., 2002). The subsequent Salvador Declaration on Open Access, released just four years later, sought to address these criticisms, underscoring the need for a more inclusive and equitable approach (SciELO, 2015). As BOAI marked its 20th anniversary, the discourse has increasingly shifted towards recognizing and addressing structural inequalities within open access. Our deflection, especially on definitions, situates within this significant tradition of critique and reassessment, acknowledging that redefining OS involves engaging with complex, longstanding debates.

A revised, expanded and ameliorated definition of OS could help preventing catastrophic consequences of unharnessed disruptive technologies, such as the one envisioned above, while also addressing some relevant concerns already discussed in literature (Düwell, 2019). Beyond being a set of practices, from open protocols to open dissemination/education, aimed at making scientific knowledge accessible to everyone, OS’s practices converge towards the goal of making scientific and technological progress possible by the implementation of scientific knowledge that is (or should be) available and accessible to everyone. OS is therefore *a collective effort to make the world more just, intending ‘justice’ as fairness* (Rawls, 1985, 2005)*, by democratizing scientific progress.* As noted by Potter, one of the founding fathers of bioethics, “biological wisdom (indeed, all wisdom) is a form of knowledge, “the knowledge of how to use knowledge for the social good”” (Potter, 1988).

The definition of OS we propose is deeply intertwined with Potter’s work because of this notion of wisdom: disruptive technologies require us to be wise in their use, and on that wisdom depend “the survival and improvement of the human species” (Potter, 1988). The strategy envisioned by Potter involved the development of a ‘third bioethics’, global and interdisciplinary, based on “a re-examined medical bioethics combined with a responsibility-oriented ecological bioethics” (Potter, 1988). However, Potter's vision of a third bioethics has largely been overlooked within the bioethics discourse, which has continued to focus primarily on medical ethics. Environmental ethics, which Potter sought to integrate, has often developed independently, highlighting a missed opportunity for a more holistic approach. Potter’s horizon is what can complement, inform, and sustain OS in its transition—or rather, in its maturation—from a set of practices to *a collective effort to increase justice, intending ‘justice’ as fairness, by democratizing scientific progress.*

It is clear that this definition of OS relies on a couple of assumptions with which we will not engage for the time being, but on which it is crucial to reach consensus (at least within liberal democracies) due to their pivotal role in this line of reasoning—this is rather a hook and a teaser for future work of political philosophers and social scientists, i.e. that we have a shared understanding of ‘progress’; and that we have a shared understanding of ‘fairness’.

### How Open Should OS Be?

Based on this horizon and on the previous considerations on disruptive technologies: how open should OS be? Shall ‘potentially problematic knowledge’ produced at the intersection between disruptive technology and academic research be publicly available under OS principles, despite the risks of dual use (as exemplified by the concrete cases we discussed)? Shall we (i.e.: the academic community) define limits to what we should be allowed to study—as long as the methods of the enquiry do not violate any established research ethics tenet? Likely not—also because such a scenario would put us in a rather uncomfortable *‘quis custodiet custodes’*^1^ type of situation. Shall we define conditions—or at least guidelines—defining how to make science open and therefore publicly available? This could be advisable. With a similar approach, white-hat hacking played a crucial role in improving security standards, and it did so by infringing security protocols and exposing their flaws (Levy, 2010). OS approaches, governed by a specific ethics, can be a part of the solution, rather than a part of the problem (Urbina et al., 2022): industries developing these technologies had, and still have, a concrete and immediate interest in fixing these flaws quickly and effectively, an interest which pivots mainly on product reliability, and on the risk of reputational damage.

Academic communities can and should be the white-hat hackers that challenge and improve the development of innovation-driven processes, whether these originate within academia or outside of it. That said, many academics might currently see their primary role limited to generating knowledge through publications. Therefore, an ethically framed OS approach, which encompasses considerations beyond the democratization of knowledge, has the potential to transform this limited perception of the academic role. By promoting transparency, collaboration, and accessibility, OS can inspire more scientists to recognize and act upon the ethical and moral dimensions of their work. This approach can significantly contribute to the democratic governance of disruptive technologies, as it encourages a culture where the pursuit of knowledge is intrinsically linked to societal well-being.

Innovation, even OS innovation, should never be considered ‘good’ per se, just because of its being ‘new’: while novelty is recognized as an innate psychological need, and therefore as a powerful drive (González-Cutre et al., 2016), the concept of novelty is inherently neutral, from an ethical standpoint. This is why the conception that ‘novel equals good’ should be tested whenever a new technology emerges, identifying its limits, and evaluating whether it fits within a model of ‘desirable future’.

### OS Ethics, Beyond ‘as Open as Possible’

The ethical issue at the very core of OS work on disruptive technologies detailed above requires defining a specific ethical framework to orient OS work beyond the ‘as open as possible’ tenet. What’s the purpose of openness? Is openness a value per se, or rather a mean to pursue other morally significant values? Defining which kind of ethical horizon should be incorporated at the intersection between OS, disruptive technologies, and democratic governance of technology is no trivial effort. First and foremost, what should be the aims of such an ethical framework?

We argue that such a horizon should be grounded on three main ethical pillars: the prosecution of the human species, the amelioration of the human condition, and the democratization of technology and knowledge. OS work related to disruptive technologies should therefore be considered acceptable if and only if it respects these three principles.

Of note, these pillars, or general ethical principles, build on each other (e.g.: there cannot be any amelioration of the human condition if the human species disappears), but they should be considered at the same level of importance when assessing whether OS work on disruptive technology is ethically acceptable. This is no case for balancing—if OS work on disruptive technologies does not satisfy even just one of these principles, it should not be pursued.

#### Prosecution of the Human Species

The first pillar requires an ecosystemic approach, a priori of any eventual considerations on important topics (but only loosely related to the present discussion) such as the inherent value of non-human life, or of ecosystems: the dependence of human existence on natural ecosystems has been widely ascertained (Alberti et al., 2003; Isbell et al., 2017). Therefore, the shared understanding of ‘progress’ must go beyond anthropocentrism and rely on ecosystemic perspectives. To pursue a bio-centric future (bio-centric in an inclusive sense, as opposed to ‘anthropocentric’ or ‘technocentric’) (Potter, 1996) we need to develop a reliable and polyphonic account of what a desirable bio-centric future should look like. Bioethics was born to incorporate input and knowledge from medicine, psychology, anthropology, philosophy, law, and theology, among other disciplines: leveraging on Potter’s notion of ‘third bioethics’ we need such an interdisciplinary effort to define where we want to go—as species and as elements of intertwined ecosystems.

Disruptive technologies can play a critical role in dooming, or in ensuring, the survival of the human species. An example: radioactive isotopes such as metastable technetium-99 are crucial for nuclear medicine, in routine applications for radiopharmacy or molecular imaging (Alberto & Nadeem, 2021); however, its production requires large amounts of highly enriched uranium (HEU). HEU target waste, stored in large amounts in production facilities, can be converted into metal, and potentially used to produce nuclear weapons, “using the well-known PUREX process, which chemistry graduate students should be capable of handling” (Hansell, 2008). “Converting this material to a weapon would not require elaborate shielding and could be performed in a garage with minimal dose to the processors” (Vandegrift et al., 2008). The PUREX process, while not originally developed within an OS framework, has been extensively published and thus made accessible, illustrating how the dissemination of scientific knowledge, whether through open or traditional means, can itself lead to dual-use concerns.

OS work on disruptive technologies can help ascertaining the alignment with the characterization of a bio-centric future by ensuring that the transformative nature of openness promotes not only accessibility but also the ethical stewardship of such technologies. By fostering transparency and collaborative oversight, OS aims to ensure that future-aligned disruptive technologies are scalable, accessible, and globally effective in tackling critical issues (Cartwright, 2009). This approach helps to mitigate the risks associated with dual-use technologies and supports the global efficacy (Cartwright, 2009) of beneficial innovations.

#### Amelioration of the Human Condition

Disruptive technologies have undeniably been transformative forces in improving the human condition, offering solutions to some of the world's most pressing problems. Mass computing has democratized access to information, making it possible for people to learn and acquire knowledge regardless of their location or socioeconomic status. Technology has revolutionized healthcare, making it possible for people to receive medical treatment remotely and access life-saving resources more easily. It has also provided tools for communication and collaboration, allowing people to connect with others across the world and work together to solve global problems. Furthermore, technology has increased efficiency and productivity in many industries, leading to economic growth and job creation. Beyond ‘barely’ ensuring the prosecution of the human species, disruptive technologies can and should help ameliorating the human condition. A shared account of what would represent an amelioration of the human condition does exist, to some degree, condensed and represented by the 17 UN sustainable development goals, and by the 2030 agenda for sustainable development (United Nations General Assembly, 2015).

OS can play a significant role in keeping the rudder on the right course: OS encourages transparency in the research process, which can help prevent unintended consequences of technological progress. By making research data and methods openly available, scientists can better understand the potential risks and benefits of new technologies, and policymakers can make more informed decisions. By making research data and methods openly available, other scientists can reproduce and verify findings, leading to a more robust understanding of the technology. Finally, some OS practices (such as citizen science or open dissemination) promote public engagement in the scientific process. By involving an informed public in decision-making, policymakers can ensure that new technologies are developed with consideration for ethical and social implications, which must be carefully evaluated and addressed to ensure that technology continues to serve as a tool for improving the human condition, rather than a means for exploitation or harm.

#### Democratization of Technology and Knowledge

The third pillar is deeply intertwined with the second. The ameliorations of the human condition fostered by technological progress should be democratized because it has the potential to provide benefits to society as a whole, or to deepen existing injustice (Feenberg, 1999; Veak, 2000): when access to technological progress is limited to a select few, the benefits of these advancements are unfairly concentrated among a small group, rather than being distributed more widely throughout society, contributing to injustice (Friedman, 1999). Moreover, democratizing access to technological progress can help to promote innovation and economic growth: by giving more individuals access to the tools and resources necessary to innovate and create, we can foster a more dynamic and inclusive innovation ecosystem (Gardner, 2011; Powell, 2012).

OS practices can play a critical role in ensuring the democratization of technology and knowledge in the context of disruptive technologies. By promoting transparency, collaboration, and the sharing of knowledge and data, OS can help to break down traditional barriers to access and facilitate the widespread adoption and use of (aligned) disruptive technologies. Overall, OS practices can help ensure that disruptive technologies are developed and used in ways that benefit society as a whole and help to ensure that the benefits of these technologies are shared broadly and fairly, rather than being limited to a select few.

### OS Ethics, Operational Principles

To help implementing the three general ethical principles detailed above throughout the OS pipeline, we propose a set of operational principles addressed to researchers, research institutions, funders, policymakers and regulators willing to engage in OS practices.

#### Researchers

1. Conduct thorough risk assessments to identify potential risks and impacts on the human species and ecosystems before initiating any research project involving disruptive technologies.
2. Ensure that work on disruptive technologies is aligned with the 17 sustainable development goals defined by the United Nations, evaluating potential long-term direct and indirect impacts on societies and cultures, involving diverse stakeholders in the process to promote inclusivity and transparency (research co-design). This alignment should be reflected in the research goals, methodologies, and potential impact assessment.
3. Actively promote open access to research findings, methodologies, and data. This includes publishing in open access journals and sharing research outputs through repositories, but should extend to the participation in science communication activities and open teaching.

#### Research Institutions

1. Enforce the assessment of research which could be conducted with an OS approach for its alignment with the three general principles, and based on the results, communicate to the research team whether their projects are suitable for OS dissemination. This could be done by existing institutional review boards (IRB).
2. Foster collaborations with local communities, NGOs, and governmental organizations, serving as hubs for interdisciplinary and participatory research, knowledge exchange, and collaboration to contribute directly to the amelioration of the human condition by engaging in community-driven research, participatory projects, citizen science, and research co-design.
3. Encourage researchers to publish their work in open access journals and share protocols, datasets, software, and any other intermediate output via open repositories, ensuring that knowledge generated from research is freely available to the public. This could be achieved by including intermediate output in scientific output evaluations. In parallel, employ personnel specifically trained in outreach and science communication to train and help researchers in crafting research findings into messages which crafted to be engaging and accessible for the public.

#### Research Funders

1. Prioritize funding for research projects that demonstrate a commitment to sustainability and responsible innovation (e.g. via IRB assessments). Funding decisions should consider potential risks and impacts on the human species and ecosystems, and support projects that aim to minimize these risks. As several public research funding schemes pose open access dissemination as a requisite for funding, research projects infringing the three general principles can still be funded, but their results should not be disseminated with OS approaches.
2. Prioritize research projects aligned with the 17 sustainable development goals defined by the United Nations, and encourage funding for translational research that focuses on translating scientific discoveries into practical applications and interventions with a potential for real-world impact and positive societal outcomes.
3. Allocate funding resources to bridge the research disparities between developed and developing regions, supporting collaborative projects that facilitate knowledge exchange, capacity building, and technology transfer to promote global research equity.

#### Policymakers and Regulators

1. Develop and enforce regulations that ensure the responsible development and deployment of disruptive technologies, such as mechanisms for monitoring and regulating the use of disruptive technologies, to ensure that any eventual pitfalls, vulnerabilities, and flaws are publicly known, as well as potential fixing strategies.
2. Encourage ongoing dialogue and debate about the ethical implications of disruptive technologies to promote responsible innovation and progress, on the notion of ‘bio-centric desirable future’ and on sustainable development goals.
3. Implement mechanisms to engage the public in policy-making processes related to disruptive technologies. Conduct public consultations, involve stakeholders in decision-making, and consider public opinion and concerns when shaping policies and regulations.

Currently, adherence to these principles is inconsistent. Many research projects do not undergo thorough risk assessments or align with the 17 UN sustainable development goals, and open access publishing is still not universally practiced. Research institutions often lack structured programs to foster community collaborations, and funders may not always prioritize sustainability and responsible innovation. Policymakers and regulators frequently face challenges in implementing and enforcing comprehensive regulations for disruptive technologies.

To illustrate this, consider a research project developing a new AI algorithm for medical diagnostics. Using these operational principles, researchers would first assess potential risks and impacts on patients and healthcare systems. The institution would review the project's adherence to ethical principles before approving OS dissemination. Funders would prioritize this project if it demonstrated sustainability and responsible innovation. Policymakers would then develop guidelines to regulate the AI’s deployment, ensuring ethical approaches are considered. Conflicts between principles, such as balancing open access with privacy concerns, would require a nuanced approach, prioritizing transparency while safeguarding sensitive information. Ongoing dialogue and stakeholder involvement would be key to resolving such conflicts.

**Fig. 1:**
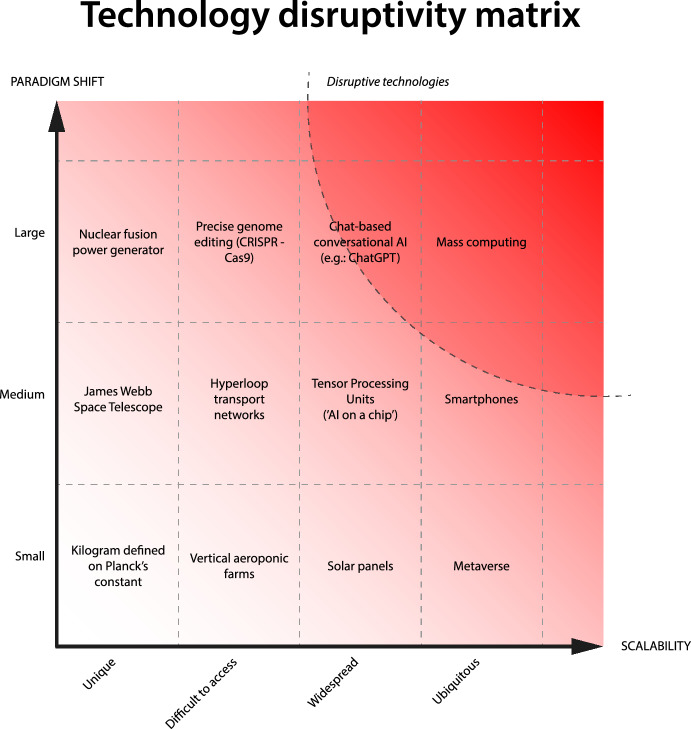
Technology disruptivity matrix. The X axis represents scalability; the Y axis represents paradigm shift. Technologies characterized by high scalability and causing a large paradigm shift are to be considered as disruptive

The operational principles outlined in this document primarily address ideal scenarios where it is evident from the outset of a research project whether it carries the potential of generating knowledge or technology with disruptive potential not aligned with the three general principles. However, disruptive technologies that are not in alignment with these principles may also arise as ‘incidental findings’ during research processes that initially appear innocuous. In such instances, the application of the operational principles and the safeguards they provide would be challenging. Consequently, it is important that researchers receive comprehensive training in research ethics that encompasses an understanding of the three general principles discussed earlier. This will equip individual researchers with the necessary tools to make informed decisions and assume responsibility when confronted with ‘non-aligned incidental disruptive technologies’, enabling them to determine whether the findings should be disseminated through OS practices—or if dissemination should be withheld altogether (Fig. 1).

## Conclusion

In this paper we problematized on the topic of ‘disruptive technology’, provided a definition of the concept, and reflected on the possibility and on the risks of doing OS work on disruptive technologies, surpassing the ‘as open as possible’ tenet. To do so, we propose an ethical framework based on a ‘third bioethics’ perspective, comprising three general principles and a set of operational principles directed to researchers, research institutions, funders, policy makers and regulators. OS practices should be built upon three ethical pillars: promotion of the prosecution of the human species, amelioration the human condition, and democratization of technology and knowledge. When a knowledge production activity violates one of the three general principles, it should not be pursued with an OS approach.

Achieving these goals requires careful regulation, especially when it comes to disruptive technologies, which can have profound effects on society, both positive and negative. While OS can promote the development of new technologies that ameliorate the human condition, it can also enable the development of applications that have unintended consequences or negative impacts. We conclude that ‘open’ should not mean neither ‘undirected’ nor ‘unregulated’, and that the openness of OS should be viewed as a means to an end, rather than an inherent ethical value.

## Footnotes

1 Who watches the watchmen?
